# Flexible network community organization during the encoding and retrieval of spatiotemporal episodic memories

**DOI:** 10.1162/netn_a_00102

**Published:** 2019-09-01

**Authors:** Amber M. Schedlbauer, Arne D. Ekstrom

**Affiliations:** Neuroscience Graduate Group, University of California, Davis, CA, USA; Center for Neuroscience, University of California, Davis, CA, USA; Neuroscience Graduate Group, University of California, Davis, CA, USA; Center for Neuroscience, University of California, Davis, CA, USA; Department of Psychology, University of California, Davis, CA, USA; Department of Psychology, University of Arizona, Tucson, AZ, USA

**Keywords:** Community detection, Episodic memory, Functional connectivity, Graph theory, Networks

## Abstract

Memory encoding and retrieval involve distinct interactions between multiple brain areas, yet the flexible structure of corresponding large-scale networks during such memory processing remains unclear. Using functional magnetic resonance imaging, we employed a spatiotemporal encoding and retrieval task, detecting functional community structure across the multiple components of our task. Consistent with past work, we identified a set of stable subnetworks, mostly belonging to primary motor and sensory cortices but also identified a subset of flexible hubs, mostly belonging to higher association areas. These “mover” hubs changed connectivity patterns across spatial and temporal memory encoding and retrieval, engaging in an integrative role within the network. Global encoding network and subnetwork dissimilarity predicted retrieval performance. Together, our findings emphasize the importance of flexible network allegiance among some hubs and the importance of network reconfiguration to human episodic memory.

## Author Summary

The degree to which task-related functional connectivity patterns remain stable or are dynamic when people learn and remember information remains largely untested. We investigated this issue by collecting fMRI while participants performed a memory encoding and retrieval task. Our results suggested that subnetworks are dynamic and tend to fragment relative to a resting-state network partition. From these changes in connectivity, we identified a subset of “movers,” or in other words, nodes that changed their allegiance to subnetworks across all aspects of the task. These findings emphasize that memory is a dynamic process involving changes in task-related functional connectivity across the brain.

## INTRODUCTION

An important goal of cognitive neuroscience is to understand how memory-related brain areas, like the hippocampus and parietal and prefrontal cortices, interact during memory encoding and retrieval (King et al., 2015; Rugg & Vilberg, 2013; Schedlbauer et al., 2014). Much of the research dedicated to episodic memory has focused on the activity within these specific brain regions by using functional magnetic resonance imaging (fMRI) (Cansino et al., 2002; Kim, 2010; Paller & Wagner, 2002; Spaniol et al., 2009), yet local activation patterns themselves cannot inform global interactions between different brain areas or corresponding functional networks. Other studies have employed resting-state fMRI to extract groups of interacting regions based on their functional connectivity patterns in the absence of stimuli; these subnetworks, in turn, have been related to memory behaviors (Andrews-Hanna et al., 2014; Tambini et al., 2010). One strength of resting-state fMRI and resting-state networks (RSNs) is that such networks have been repeatedly and robustly identified over many participants and studies (Beckmann et al., 2005; Damoiseaux et al., 2006; Fox et al., 2006; Power et al., 2011; Smith et al., 2009; Yeo et al., 2011), and they show meaningful relationships to memory performance more generally (Vaidya & Gordon, 2013). While there is little question that RSNs are remarkably robust both within and across participants and even for different forms of resting (e.g., Golland et al., 2007), the lack of explicit tasks during this state limits inference about the engagement of shorter time-frame changes directly relevant to memory encoding and retrieval.

A common finding from studies of task-related functional networks is that they deviate somewhat in structure compared with those derived from resting state (Bolt et al., 2017; Cohen & D’Esposito, 2016; Keerativittayayut et al., 2018; Spadone et al., 2015). Furthermore, some studies suggest that functional subnetworks, and even single regions within those subnetworks, can exhibit a large range of connectivity patterns over time (Braun et al., 2015; Inman et al., 2017) and over different tasks (Bassett et al., 2011; Cohen & D’Esposito, 2016; Spadone et al., 2015). Hence, past findings indicate that flexibility in functional connectivity between RSNs and task-related networks and between different task states may be pertinent to behavior. In particular, a limited number of studies have employed task-related functional connectivity analyses to better understand such memory-related changes (Geib et al., 2015; Inman et al., 2017; Keerativittayayut et al., 2018; King et al., 2015; Westphal et al., 2017). However, few studies have looked specifically at how task-related networks change as a function of episodic memory encoding and retrieval, and those studies that have did not investigate changes in task-related network configurations and subnetwork structure during different aspects of memory.

Previous work from our lab has provided evidence for anatomical (Copara et al., 2014; Ekstrom et al., 2011; Schedlbauer et al., 2014) and spectral (Watrous et al., 2013) differences between the *retrieval* of spatial and temporal contextual information related to episodic memories (i.e., the “when” and “where” of an event). Specifically, Schedlbauer and colleagues (2014) showed substantial overlap between networks derived from the retrieval of these different contexts but also showed differential anterior and posterior subnetworks related to the processing of temporal and spatial information, respectively. These findings would suggest that a stable core network might underlie mnemonic cognition, but there is relevant variability in regional participation and interactions during contextual processing, in which both these stable and flexible connectivity patterns can be captured using large-scale network representations. These results are echoed in another study in which the authors found additional variance in connectivity, or flexibility, when describing a stable core and flexible periphery whose characteristics predicted learning success (Bassett et al., 2013). However, it is not clear how that stable core corresponded to RSNs delineated by previous studies and how they related specifically to memory processing.

One way to identify a specific group of nodes within a network, such as a stable core, is to examine the modular or community structure. Community detection algorithms are frequently employed to partition the brain into nonoverlapping sets of regions, termed subnetworks, communities, or modules (Sporns & Betzel, 2016) in order to make inferences about cognition (e.g., Smith et al., 2009). Typically, such analyses focus on communities that are more distinct or “modular” and are defined as either 1) having a larger number of *intra*modular connections and fewer *inter*modular connections that link to other modules or 2) are independent components. RSNs are usually defined in this manner. One possibility, which we explore here, is that deviation from RSN architecture during different task states detected via altered community structure may be informative in understanding the functional organization of large-scale networks and the functional role of subsets of nodes during mnemonic processing.

The current study performed a detailed analysis of task-related fMRI connectivity patterns during a source memory paradigm, in which we recorded both the learning (encoding) and remembering (retrieval) stages of item-context associations (Cansino et al., 2002; Mitchell & Johnson, 2009). Using the techniques of graph theory, we probed network organization over four different cognitive states: encoding, retrieval, and the different contexts under which encoding and retrieval occurred (in our case, spatial versus temporal). To determine variable subnetwork identities (i.e., changes in composition and connectivity of groups of regions), we first performed a data-driven partition and also used a resting-state derived partition based on the Power Atlas (Power et al., 2011), a robust atlas widely applied in other neuroimaging studies (Cole et al., 2014; Power et al., 2013). We directly compare these two partitions during the four different components of the memory task as well with the partitions derived from an “active baseline” task (Stark & Squire, 2001). Finally, we modeled how much variance in retrieval performance could be accounted for by the data-driven and resting-state partitions. Together, our findings suggest that memory encoding and retrieval involve some degree of flexible brain-wide reconfigurations that are relevant to retrieval performance and that those more flexible (rather than stable) brain areas assume an integrative role within the networks.

## MATERIALS AND METHODS

### Participants

Twenty-four young, healthy adults were recruited from the University of California, Davis and from the surrounding communities to participate in the experiment (mean age = 23.4 years, range = 18–33, 12 women). Eighteen participants were included in the final analyses as three participants were excluded due to technical problems during scanning, one due to excess head motion, one due to a possible incidental finding, and one due to performance accuracy falling outside two standard deviations from the mean group accuracy (mean age = 23.8 years, range = 18–33, 10 women). All participants were right-handed, had normal or corrected-to-normal vision, and were screened for neurological disorders. The experiment was approved by the Institutional Review Board at the University of California, Davis. All participants provided written informed consent and were compensated $10 per hour.

### Experimental Design and Procedure

Participants underwent a practice version of the task and then encoding and retrieval sessions. During the preparatory practice session (approximately 15 minutes), participants generated a primarily spatial (a picture of the layout of their current residence from an overhead perspective) and a primarily temporal (a timeline of 10–15 memories from throughout their lives) context, which were then used during the encoding session. Participants were told to include memories that they could clearly visualize in their mind’s eye and readily order, although they were not specifically required to use highly significant or emotionally salient memories. Once both contexts were complete, participants had 5 minutes to review the locations and events before starting the experiment (they did not have access to the context sheet during the experiment).

Immediately following the preparatory session, participants entered the scanner for the encoding and retrieval sessions. Using an event-related paradigm, participants were shown 80 objects in four runs of 20 objects each. Stimuli consisted of 100 color photographs of unique objects against a white background, which were drawn from the CVCL stimulus set (Brady et al., 2008). Each run alternated between spatial and temporal, and the order was counterbalanced across participants. Each trial began with a two-second cue period in which the instruction “Imagine” and an object image appeared on the screen. After 1 second, a context cue (“in SPACE” or “in TIME”) appeared below the object image, indicating whether the object should be mentally “placed” in their spatial or temporal contexts. The complete cue remained on the screen for an additional second and was replaced by a central fixation cross for 4 seconds. Participants were instructed to visualize the cued object in the cued context during the fixation period.

For spatial trials, participants imagined the object somewhere within the spatial layout of their residences, focusing on where the object was in relation to other elements. For temporal trials, participants were instructed to imagine the object somewhere along their memory timelines in one of the selected events. For more details and validation of the behavioral paradigm, please see Bouffard et al. (2018). Following the fixation period, participants had 6 seconds to rate how vividly they had imagined the object in context on a 1 to 4 scale, with “1” indicating that they were unable to imagine the item in context and “4” indicating that they were able to clearly imagine the item in context with lots of detail. Each trial lasted for 12 seconds total. Between trials, presentation of stimuli was jittered using a central fixation cross with an intertrial interval of 1, 2, or 3 seconds. At the end of each encoding run, once all 20 trials had been presented, participants performed a distractor task lasting 1 minute; participants pressed “1” when an X and “2” when an O appeared on the screen. Specifically, this “active” baseline task is frequently employed in memory-related tasks because it involves the least amount of activation in memory-related areas compared with tasks like fixation (Stark & Squire, 2001).

**Figure 1.**
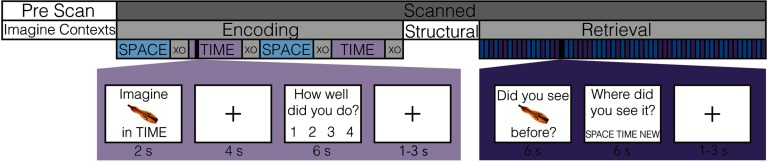
Experimental paradigm. Participants completed a source memory experiment consisting of prescan, encoding, and retrieval sessions. During the encoding session, participants “placed” a presented object (“Imagine…in TIME/SPACE”) in either their spatial (familiar spatial layout) or temporal (timeline of personal events) contexts generated during the prescan session. During the retrieval session, a series of previously presented items interspersed with novel items was shown, and the participant was required to identify the item (“Did you see…before?”) and the context in which it was placed during the encoding session (“Where did you see it?”).

After completing the encoding session, participants began the retrieval session where the 80 previously encountered objects and 20 lure objects were presented across four runs of 25 trials each. For each trial, the object image was first presented on the screen for 6 seconds; the participant was then required to make an item recognition judgment. After those 6 seconds, the participants answered a source judgment question where they had 6 seconds to indicate if the item had appeared in space, in time, or was new. Stimulus presentation was jittered in the same manner as in the encoding session, and retrieval runs were counterbalanced across participants. See Figure 1 for details. This task mirrors other tasks that have been used to study episodic memory with patients and fMRI (Cansino et al., 2002; Davachi et al., 2003; Duarte et al., 2010).

### Imaging Acquisition

Imaging data were collected using a 32-channel head coil on a 3T Siemens Skyra MR machine at the UC Davis Imaging Research Center in Davis, CA. Functional images were acquired using a whole-brain (2 × 2 × 2.2 mm voxel) multiband echo-planar imaging (EPI) sequence (slices = 52, TR = 1,600 ms, TE = 25 ms, FA = 65°, FOV = 208 mm, bandwidth = 1,550 Hz/pixel). A high-resolution T1-weighted structural 3D magnetization prepared rapid acquisition gradient echo (MPRAGE) was also acquired (1 × 1 × 1 mm voxel). Experimental stimuli were presented using PsychoPy software (Peirce, 2007), and responses were collected using an MRI-compatible button box.

### fMRI Data Processing

Functional image processing was performed using Statistical Parametric Mapping (SPM12) software (https://www.fil.ion.ucl.ac.uk/spm/software/spm12/). Functional images were corrected for differences in motion, coregistered to structural space (high-resolution MPRAGE), and segmented into gray and white matter in preparation for spatial normalization. Images were normalized to MNI space by using the DARTEL toolbox (Ashburner, 2007). Finally, functional images were spatially smoothed using a 4-mm FWHM isotropic Gaussian kernel and high-pass filtered at 128 seconds to remove scanner drift and cardiac/respiratory artifacts.

### Mnemonic Task Functional Connectivity

As we have described previously (Schedlbauer et al., 2014), we assessed inter-regional interactions throughout the brain by using a beta series approach (Rissman et al., 2004). Importantly, the beta series method allowed us to derive a network representation for different task conditions that have interleaved trials (rather than block-design) during a scanning session. Briefly, each voxel’s BOLD response in the task was modeled in a general linear model (GLM) (Friston, 1994) as an individual regressor specifying the onset of each trial convolved with the canonical hemodynamic response function (HRF). Six head motion regressors were also entered into the GLM. The parameter, or beta, estimates derived for each trial for each voxel were then sorted by condition (space, time, encoding, retrieval) into a series. Only trials where objects that were correctly identified as old were used, but all conditions consisted of both correct and incorrect source memory judgments to determine networks involved in general spatiotemporal mnemonic processes. The beta series of voxels belonging to a region of interest (ROI) were subsequently averaged culminating in 223 average beta series per condition. Each ROI consisted of a 3 × 3 × 3 voxel cube centered on the coordinates obtained from the Power Atlas (Power et al., 2011). The Power Atlas contains 264 regions total; only labeled cortical regions that were part of an established RSN were included in our analyses (total = 219).

Because of lack of coverage within the medial temporal lobe, we included four additional ROIs (left and right, parahippocampal gyrus and hippocampus) derived from the Shirer Atlas (Shirer et al., 2012) for a total of 223 nodes. These nodes comprised the memory or MEM subnetwork. Finally, we computed the Pearson product-moment correlation coefficient between all ROI’s beta series, creating a correlation matrix of all pairwise combinations describing the strength of the functional relationship between two regions for each individual. Because motion has been found to affect functional connectivity estimates (Power et al., 2012), trials that included any volume that exceeded the average frame-wise displacement threshold of 0.55 mm were excluded from the beta series. In addition, for each subject, random trials from each beta series were removed to create equal-length beta series for spatial and temporal encoding and for spatial and temporal retrieval. The mean and standard deviation of the number of trials comprising the beta series were calculated for each condition (spatial, temporal encoding: 37.22 ± 2.80; spatial, temporal retrieval: 34.11 ± 6.45).

### Baseline Task Functional Connectivity

To better compare the topology of memory task-based networks with a “null” network, we employed similar methods outlined by Fair and colleagues (2007), where residual time-courses from interleaved nontask periods can be extracted and used to simulate “continuous” resting-state data. During participant-specific first-level modeling, individual stimulus onsets from each condition were convolved with the canonical HRF and entered into a GLM. For encoding, trials were modeled for a total of 6 seconds beginning with the onset of the imagination cue. Retrieval trials were modeled with varying durations consisting of the 6 seconds of item recognition plus the response time of the source recognition question. Trials in which no response was made during the second half were modeled with a duration of 12 seconds, the total length of the trial. Intertrial intervals, consisting of a central fixation cross and the distractor task at the end of each encoding session, were implicitly modeled. In addition to task-related regressors, six head motion regressors for each session were also entered into the GLM. We then constructed a baseline network derived from the residual time series (extracted from the univariate models) during the four 1-minute distractor tasks after each encoding block. We excised ∼160 seconds of data after each block and concatenated the segments; to ensure no effects from the delay of the HRF, only time points beginning 18 seconds after the distractor task started were included in the series. The Pearson product-moment correlation coefficient between all ROI’s residual time series was computed to create the baseline task connectivity matrices.

### Network Analyses

Networks were first Fisher-transformed, and any negative edges or edges from nodes that were within 20 mm of each other were set to zero in the connectivity matrices. These short distance correlations may be suspect because they could arise due to shared local neural activity, blurring from data preprocessing methods, or head motion rather than representing genuine regional interactions (Power et al., 2012, 2013). Analyses were performed both at the individual- and group-level networks. Group connectivity matrices consisted of the average of all individual participant connectivity matrices. To understand data-driven community structure within each network, we employed the modularity maximization algorithm (known as Louvain, implemented as in Rubinov & Sporns (2010) with positive, weighted edges, in which the weights were determined by the strength of the transformed Pearson’s correlation coefficient). By setting the algorithm’s resolution parameter to 1.25, the subnetwork or community size and number would better match those of the RSNs outlined by the Power Atlas (Cohen & D’Esposito, 2016).

Because network thresholding is still a debated issue within the graph theory community, we wanted to estimate the most stable network partition across a number of algorithm iterations and across a range of thresholds by using consensus clustering methods (Lancichinetti & Fortunato, 2012). For each graph threshold (cost range: 0.01–0.99 in 0.1 increments), we ran the community detection algorithm for a total of 100 iterations to build a consensus matrix where each cell in the matrix indicates the proportion of times two modules were assigned to the same community. This matrix was then thresholded at 0.5 to reduce spurious node assignment, and the detection algorithm was applied a final time to extract a stable partition at that particular threshold. From each of these semifinalized partitions at the different thresholds, we again compiled a matrix indicating the proportion of times two nodes were assigned to the same module across all thresholds, thresholded at 0.5, and extracted a final partition. This partition became the final partition used to identify the data-driven communities or subnetworks for each network condition. (For a more in-depth discussion of different module detection techniques, see Sporns & Betzel, 2016.) BrainNet Viewer (Xia et al., 2013) was used to visualize the various networks and anatomical distribution of nodes.

### Graph Theory Metrics

**Figure 2.**
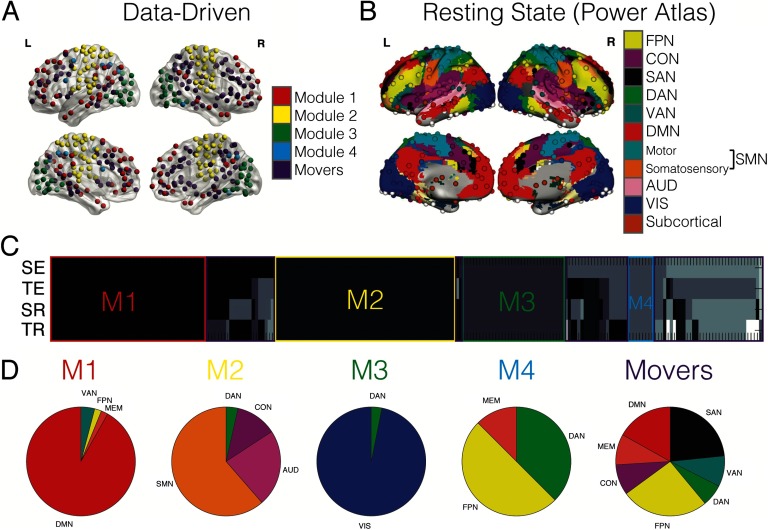
Data-driven community detection compared with RSNs. (A) The anatomical distribution of nodes used in the analyses is shown on the transparent brains (left and right, lateral and medial views). The color of the node indicates to which data-driven module each node belongs. (B) The anatomical distribution of nodes extracted in Power et al. (2011) with each resting-state network (RSN) indicated in the different colors (Figure from Cole et al., 2013). FPN = fronto-parietal; CON = cingulo-operculo; SAN = salience; DAN = dorsal attention; VAN = ventral attention; DMN = default mode; SMN = sensorimotor; AUD = auditory; VIS = visual. (C) The gray-scale matrix (4 conditions × 223 nodes) indicates the module identity for each node for each group-level network. SE = spatial encoding; TE = temporal encoding; SR = spatial retrieval; TR = temporal retrieval. Nodes belonging to the same module are the same color, resulting in four stable data-driven modules outlined in red, yellow, green, and blue. M1 = Module 1; M2 = Module 2; M3 = Module 3; M4 = Module 4. Any node boxed in purple is considered a flexible “mover” node, not belonging to a stable module. (D) Each pie chart shows the proportion of nodes from the RNSs that now belong to each data-driven module.

To examine global patterns in network topology, we vectorized connectivity matrices and correlated them between conditions (e.g., space encoding and temporal encoding), giving a single value or similarity assessment. In addition to community structure, we employed several other metrics by using the Brain Connectivity Toolbox (Rubinov & Sporns, 2010). Both within-module degree centrality and participation coefficient (PC) were used to assess local node flexibility. Importantly, the with-module degree centrality was calculated for the modules obtained from that network’s data-driven partition, not the modules identified across all four conditions, as in Figure 2. Within-module degree centrality is the standardized sum of weighted, undirected connections of a particular node within its own community, or total intramodular connectivity. It is defined as follows:

$$z_{i}=\frac{k_{i}\left(m_{i}\right)-\overset{-}{k}\left(m_{i}\right)}{s^{k\left(m_{i}\right)}}$$

where *z*_*i*_ is the within-module z-score of node *i*, *m*_*i*_ is the module containing node *i*, *k*_*i*_ (*m*_*i*_) is the within-module degree of *i*, and $\overset{-}{k}$ (*m*_*i*_) and *σ*^*k*(*m*_*i*_)^ are the mean and standard deviation of the within-module *m*_*i*_ degree distribution, respectively. The participation coefficient assesses the connectivity of a node to other communities, or intermodule connectivity, and is defined as follows:

$${PC}_{i}=1-\underset{m\in M}{\sum }{\left(\frac{k_{i}(m)}{k_{i}}\right)}^{2}$$

where *M* is the set of modules, and *k*_*i*_ (*m*) is the number of connections between *i* and all nodes in module *m*. Even though this metric has a range from 0–1, we z-scored the values from each network because of a narrow distribution of calculated values. To assign an identity to each node, which indicates its role in network communication, we used the within-module degree centrality and participation coefficient distributions to label each node as connector, provincial, satellite, and peripheral (Bertolero et al., 2015; Cohen & D’Esposito, 2016). Nodes important for both intra- and intermodular connectivity are considered connector hubs and have high *z*_*i*_ (≥ 0) and high PC (≥ 0). Provincial hubs are important for only intramodular connectivity and have high *z*_*i*_ (≥ 0) but low PC (< 0). Satellite nodes have low *z*_*i*_ (< 0) but high PC (≥ 0) and are important for intermodular connectivity. Lastly, peripheral nodes are sparsely connected nodes, and those few connections are not to other modules (i.e., low *z*_*i*_ (< 0) and low PC (< 0)). We adapted these *z*_*i*_ and PC thresholds, so that all nodes would be specified as one of the four identities. Normalized mutual information (NMI) is a quantitative way to compare community detection partition schemes overall; we employ this metric to assess the similarity between the data-driven and RSNs communities. Finally, we introduce the fragmentation metric to assess changes in *specific* subnetwork membership. Fragmentation quantifies the proportion of the nodes of a defined RSN that realign with other (in this case, data-driven) modules. Fragmentation, F, of RSN_*x*_ is defined as:

$$F_{x}=1-\frac{C_{x}}{T_{x}}$$

where *C*_*x*_is the largest within-module connected component of RSN_*x*_ nodes across all modules, and *T*_*x*_ is the total number of nodes in RSN_*x*_.

For statistical comparisons, *χ*^2^-test of independence and two-way analysis of variance (ANOVA) were used to determine condition and subnetwork differences for group and individual levels, respectively. Bonferroni corrections were used to correct for multiple comparisons.

### Regression Models

To assess brain-behavior relationships, we correlated overall network similarity with performance for all condition pairs (e.g., spatial and temporal encoding, spatial and temporal retrieval). Results were corrected for the six comparisons by using Bonferroni corrections with a significance threshold of corrected *p* = 0.05. As an alternative to stepwise regression for more complex regressions, the least absolute shrinkage and selection operator (lasso) regression method performs variable selection among a set of dependent variables in linear regression models by employing regularization (referred to here as the tuning parameter) (Tibshirani, 1996). To select a subset of predictors that best predicts the response variable, the algorithm can shrink variable coefficients to zero, thus producing a more parsimonious model. To determine if there were subsets of the network that could also capture the relationship between participant performance and overall network similarity, we constructed a multiple linear regression where the similarity between two subnetworks constituted each independent variable.

## RESULTS

### Experimental Paradigm and Behavior

In a prescan session, participants (*n* = 18) each generated two contexts: TIME (a series of organized personal events) and SPACE (a familiar spatial layout). Once identified and memorized, participants next completed a scanned encoding session, where they “placed” a presented object in the cued context (SPACE or TIME). After each encoding trial, they answered a vividness judgment question of how well they placed that item in that context. During a subsequent scanned retrieval session, for each trial, participants made an item recognition judgment regarding either a previously presented or newly presented object and also made a source judgment regarding the context the item was previously associated (Figure 1). The proportion of trials that participants correctly identified both the item and source context will be referred to as retrieval performance. A paired samples *t*-test revealed no significant differences in performance between the spatial and temporal source context conditions, although the difference was trending (*t*(17) = 2.08, *p* = 0.0532); there was no difference for reaction time (*t*(17) = −1.01, *p* = 0.326). Table 1 contains both accuracy and reaction time summary statistics.

**Table 1.** Behavior summary statistics: Task-related changes in community structure

**Measure**	**Space Mean**	**Space SD**	**Time Mean**	**Time SD**
Item correct	0.95	0.059	0.93	0.048
Item correct source correct	0.86	0.13	0.80	0.13
Item reaction time	1.50	0.31	1.58	0.35
Source reaction time	0.94	0.33	0.98	0.39

To address how different aspects of our task might be modularized in the brain, we first partitioned the networks by using a data-driven approach that relied on consensus clustering over a range of graph thresholds (see Materials and Methods section for details). We then compared changing topology of these data-driven, task-based networks (Figure 2A) to an established RSN partition extracted by Power and colleagues (2011) (Figure 2B). The Power RSNs consist of the fronto-parietal (FPN), cingulo-operculo (CON), salience (SAN), dorsal attention (DAN), ventral attention (VAN), default mode (DMN), sensorimotor (SMN), auditory (AUD), visual (VIS), and subcortical subnetworks. The memory (MEM) subnetwork was created from regions from the Shirer Atlas (see Materials and Methods). Figure 2C shows a matrix of community assignment. The gray-scale color indicates the assignment of each individual node to a module within each network as a function of task (spatial encoding, temporal encoding, spatial retrieval, temporal retrieval). There was considerable overlap in community partitions across the mnemonic conditions as indicated by the colored boxes in Figure 2C. In other words, sets of nodes were consistently assigned to the same community or module across all four conditions; each of these stable groups formed a data-driven module for a total of four modules (blocks of same colored nodes). One notable difference between the task-based partitions versus the Power-defined RSNs is that the data-driven network has far fewer modules, yet these modules remain relatively stable across the different subcomponents of the memory task.

The data-driven partition also revealed a subset of nodes, here termed “movers,” that were flexible and altered their community alliance across conditions (these mover nodes are surrounded by purple boxes). One of these movers included the hippocampus, an important structure for memory (Eichenbaum et al., 2007; Scoville & Milner, 1957). A detailed analysis of medial temporal lobe connectivity patterns can be found in the Supporting Information (Figure SI 1). Each data-driven module is composed of nodes that have been previously assigned to specific RSNs (as outlined by Power et al., 2011, and shown in Figure 2B). Figure 2D shows the percentage of RSN nodes that make up each data-driven module, including the mover group (see far right pie chart labeled “Movers”). Noticeably, the movers consist of nodes from multiple RSNs and do not belong primarily to one specific subnetwork. In this way, these nodes that have been previously classified as belonging to a specific RSN alter their connectivity in such a way that their community allegiance shifts, ultimately changing the composition of communities within the brain during a cognitive task. Thus, the data-driven partition revealed not only a smaller set of subnetworks but also that some of these nodes appear more variable in their allegiance as a function of memory encoding and retrieval.

One possibility is that the movers were driven by differences in univariate activation patterns rather than change in connectivity. To address this issue, we correlated the average connectivity strength of the mover nodes with the corresponding univariate activation within each ROI for each participant and each condition. Overall, we found only four significant correlations across all condition and participants (total of 72 correlations), which nonetheless were not clustered in any meaningful way (Supporting Information Figure SI 2). An additional possibility is that the mover nodes were in fact “moving” because of high degrees of variability in weak connections. By correlating the connectivity vector of each node for each pairwise combination of tasks, we found that mover nodes showed a lower correlation than the nonmover nodes (Supporting Information Figure SI 3), supporting the idea that movers are meaningfully changing their connectivity profile, rather than being an artifact of the Louvain method. Together, these analyses support the idea that movers were changing the subnetworks with which they interacted, and that such movement was not due to univariate activation confounds or variability in weak connections.

**Figure 3.**
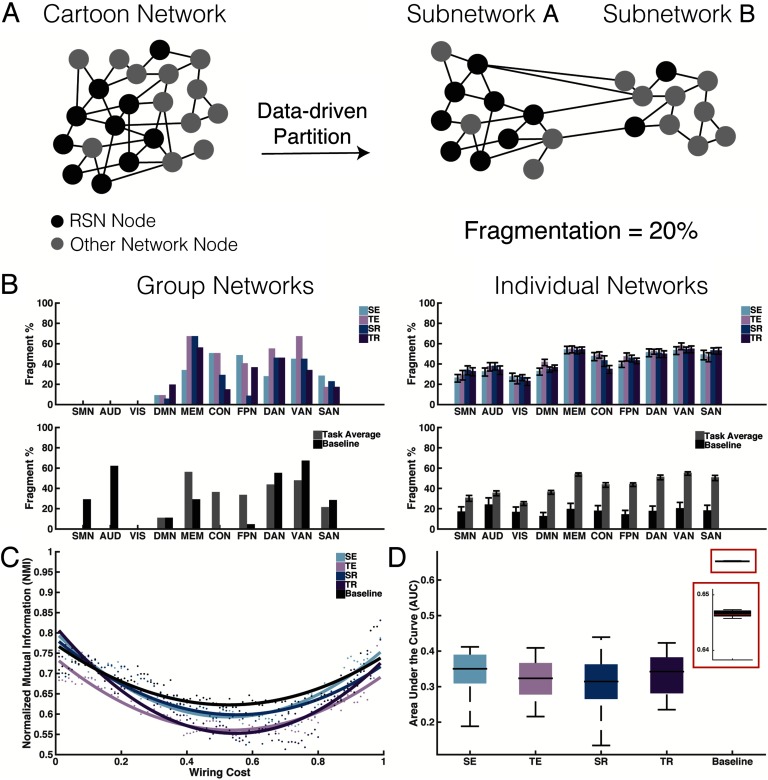
Comparison of data-driven and resting-state partitions. (A) A cartoon network consists of a subset of nodes that are considered part of a resting-state network (RSN) (black nodes). When applying a community detection algorithm (i.e., data-driven partition) to the entire network, two data-driven subnetworks are obtained. Both data-driven Subnetwork A and data-driven Subnetwork B contain nodes that were formerly labeled as RSN nodes. Because 2 of the 10 RSN nodes belong to another module, it can be said that the RSN is 20% fragmented. (B) When applying the fragmentation metric to the group-level (left panels) and individual-level (right panels) mnemonic networks (top row), a high percentage of fragmentation occurs for those higher order subnetworks compared with those associated with primary sensory areas (SMN, AUD, VIS). When averaging the fragmentation across mnemonic conditions and plotted against the baseline network (bottom row), a high percentage of fragmentation occurs overall for task compared to baseline. (C) We calculated the normalized mutual information (NMI) between the data-driven and RSN partitions for different wiring costs for the group-level, task-based networks (colored dots). For each condition, we computed a best-fit to the data (colored curves). (D) We applied the analysis steps outlined for panel C to each individual participant network, and the area under the curve was calculated. There were no group-level differences between task-based conditions, but all were significantly lower than the baseline network condition. These findings indicate that the baseline network partitions were more similar to those RSN partitions. SE = spatial encoding; TE = temporal encoding; SR = spatial retrieval; TR = temporal retrieval; FPN = fronto-parietal; CON = cingulo-operculo; SAN = salience; DAN = dorsal attention; VAN = ventral attention; DMN = default mode; SMN = sensorimortor; AUD = auditory; VIS = visual; MEM = memory.

To better quantify reorganization between subnetworks across the four conditions in our experiment, we developed a simple metric, termed fragmentation, which allowed us to directly compare how the data-driven partition assigned nodes changed community assignment relative to the RSN-delineated subnetworks. Figure 3A provides a schematic of the fragmentation concept. We applied the fragmentation computation to each RSN (a total of 10 which are shown in Figure 1B, the sensory and motor RSNs have been combined into a sensorimotor subnetwork (SMN)) with the group-level (top left panel) and individual-level (top right panel) networks for each condition of the task (Figure 3B, top row). Subnetworks associated with primary sensory functions (SMN, AUD, VIS) (Figure 3B) showed overall the least fragmentation. In contrast, those associated with higher order processes (DMN, MEM, FPN) showed the highest degrees of fragmentation. A two-way ANOVA for the individual-level networks (top right panel) showed a significant main effect of subnetwork (F_9,690_ = 31.7, *p* < 0.001) but no main effect for task or interaction effects. Post hoc comparisons of the MEM RSN showed significant differences between all other RSNs except DAN, VAN, and SAN (*p* < 0.05, Bonferroni corrected).

Because of the significant levels of fragmentation of some RSNs (as high as 66%), we wanted to ensure that this divergence from resting-state architecture was not just a product of our particular set of participants and scanning parameters. Thus, we compared the fragmentation of the task-based networks to an active baseline task commonly used in the memory literature to control for rumination. Figure 3B (bottom row) shows the fragmentation score for each RSN (averaged across the four task conditions from the left panels) and the baseline network fragmentation score. Similar to network partitions from RSNs, we again found significant main effects of both subnetwork (F_9,340_ = 3.5, *p* < 0.001) and task (F_1,340_ = 168.0, *p* < 0.001) and an interaction effect between subnetwork and task (F_9,340_ = 2.7, *p* = 0.004; Figure 3B, bottom right panel). Thus, the partition based on the null baseline task from our same recording session was more similar to the RSN partition as shown through reduced fragmentation compared with the fragmentation of memory-related partitions during encoding and retrieval. Although we observed some differences in individual versus group fragmentation patterns, suggesting that individual patterns may be more variable than what is common across individual networks, the patterns for greater fragmentation among cognitive versus sensory subnetworks held. These data suggest that episodic memory induces higher degrees of fragmentation than an appropriately compared “null” baseline. From this perspective, community structure is flexible compared to, and even between, tasks that vary only along a single dimension, like encoding context.

To better understand how network partitions change as a function of network connectivity cost, we applied NMI over a range of thresholds, which shows more generally how similar two partitions (RSN versus task-data-driven and null-data-driven) are to each other (Figure 3C). NMI values of one and zero indicate identical versus completely dissimilar partitions, respectively. We applied polynomial functions fit to the group-level data for each task and baseline conditions to compare thresholding patterns (Figure 3C and D). The NMI over a range of thresholds was computed for each individual, and the area under the curve (AUC) was calculated to derive a single metric. A one-way ANOVA showed a significant effect of condition on AUC (F_4,85_ = 118.6, *p* < 0.001) with post hoc comparisons revealing significant differences between baseline and the other task conditions (*p* < 0.001, Bonferroni corrected) (Figure 3D). Thus, compared with the task-based network partitions, the baseline network partitions were more similar to the RSN partitions. However, qualitatively, the task-based partitions were more (or less) like RSN partitions depending on the sparsity of the network (see “U-shaped” curve). Together, these results highlight that differences in network community structure from RSNs are contingent on external demands like task type and, to a lesser extent, analytic choices like wiring cost. Despite some of these differences (like function of wiring cost), task-related fragmentation remained significantly above that of the null baseline.

### Node Flexibility and Identity Across Communities and Task

**Figure 4.**
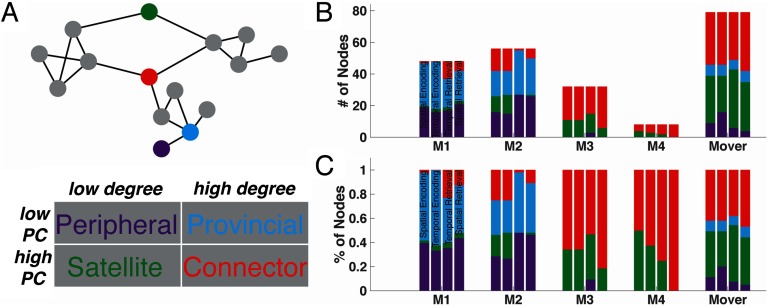
Node identities across data-driven modules and mnemonic conditions. (A) A cartoon network shows three distinct groups of nodes (i.e., modules or communities) that are connected to each other by the red and green colored nodes. Nodes that have an integrative role and connect communities have a high participation coefficient (PC). Highly connected nodes (e.g., red and blue nodes) have a large number of edges and are defined as having a high degree centrality. Nodes important for both intra- and intermodular connectivity are considered connector hubs and have high degree and high PC (e.g., red node). Provincial hubs are important for only intramodular connectivity and have high degree but low PC (e.g., blue node). Satellite nodes have low degree, but high PC, and are important for intermodular connectivity (e.g., green node). Lastly, peripheral nodes are sparsely connected nodes, and those few connections are not to other modules (i.e., low degree and low PC) (e.g., purple node). The table below the cartoon network identifies these different node types. (B) Every node in the network was assigned an identity based on the four definitions, and the number of node types for each data-driven module is plotted. Each of the bars for each module corresponds to the four conditions (spatial encoding, temporal encoding, spatial retrieval, temporal retrieval). (C) This plot shows the same data as Panel B, but rather than the number of nodes, it shows the percentage of nodes. M1 = Module 1; M2 = Module 2; M3 = Module 3; M4 = Module 4.

Our findings thus far suggest that data-driven partitions of task-based fMRI during memory encoding and retrieval reveal both stable groups of nodes that reflect particular primary-order RSNs and “mover” nodes that deviate from those communities. An additional important question regards the role of these “mover” nodes and the extent to which different nodes might serve as areas of important communication within and between networks (i.e., “hubs”). Nodes with high degree and a large number of connections to other modules (i.e., a high participation coefficient) are considered connector hubs, whereas those high degree but low participation coefficient nodes are defined as local or provincial hubs (Rubinov & Sporns, 2010). Sparsely connected nodes are labeled as peripheral and satellite nodes if they have low and high participation coefficients, respectively. Please see Figure 4A for a detailed depiction of each node type. These node labels provide important insight into how community structure might change as a function of task and help better characterize regions that are important for global and local network communication and function within and across these different communities.

**Figure 5.**
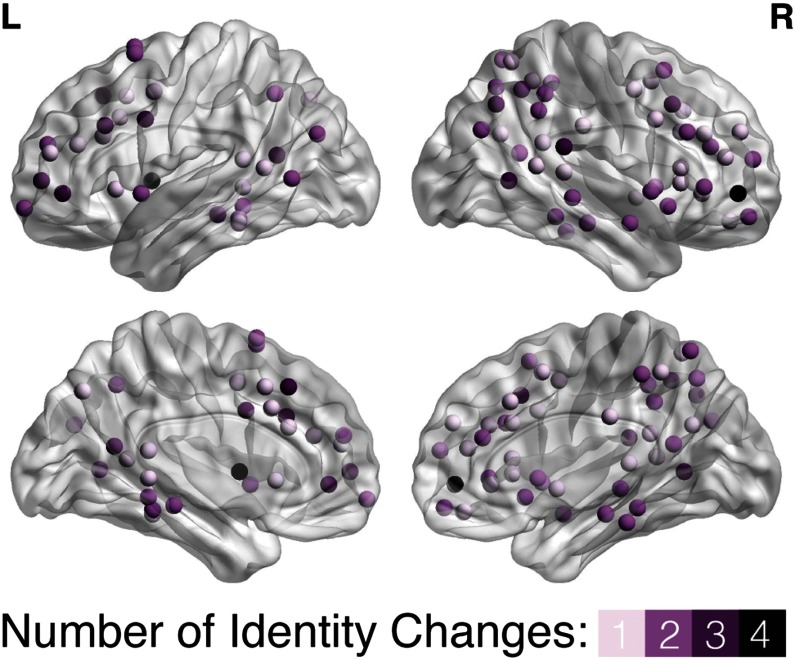
Number of times a node changes identity. The anatomical distribution of mover nodes plotted on the transparent brain (left and right, lateral and medial views) shows the number of times a mover node changes node identity (defined in Figure 4) across the four mnemonic conditions (spatial encoding, temporal encoding, spatial retrieval, temporal retrieval).

For each module in the data-driven community partition from fMRI taken during encoding and retrieval, Figure 4B shows the nodal composition, or the identities of the nodes, based on the four node types outlined in Figure 4A for each condition (each a separate bar). Rather than the number of nodes in each module, Figure 4C, similar to Figure 4B, shows the percentage of each type of node in each module across the four conditions. We conducted an omnibus *χ*^2^-test of independence to examine the relation between node identity and module (we averaged across the four conditions because the distribution of node types was similar across them), finding a significant difference between the variables (*χ*^2^(15) = 117.4, *p* < 0.001). The “mover” nodes (in addition to Modules 3 and 4) had higher numbers (and percentages) of connector hubs compared to Module 1 (*χ*^2^ of number of connector nodes: (*χ*^2^(1) = 30.3, *p* < 0.001) and Module 2 (*χ*^2^ of number of connector nodes: (*χ*^2^(1) = 29.4, *p* < 0.001), suggesting a larger integrative role for mnemonic processes. The anatomical distribution of the mover nodes indicates that the different node types are spread across the brain rather than clustering in a specific location, with node identity changing across conditions (Figure 5). Please see Supporting Information Table 1 for a list of nodes belonging to each data-driven module and their identity for the four mnemonic conditions.

### Memory-Evoked Functional Connectivity Patterns and the Relationship with Performance

**Figure 6.**
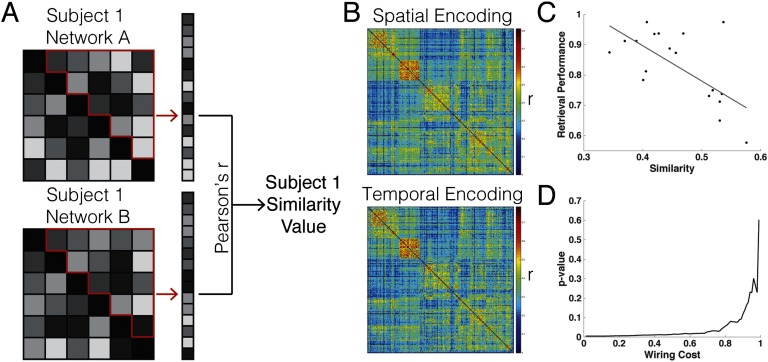
Node identities across data-driven modules and their relationship to task performance. (A) Schematic shows a theoretical Subject 1’s connectivity matrices for two conditions A and B. The unique values in the matrix (outlined in red) are vectorized and correlated with each other using Pearson’s *r*. This produces a single value metric, called similarity, to assess how close the global connectivity patterns are across the network. (B) The group-level connectivity matrices for the spatial encoding (top panel) and temporal encoding (bottom panel) are shown. (C) Each individual’s similarity value between the spatial encoding and temporal encoding networks (both unthresholded) were plotted against their retrieval performance, resulting in a significant negative relationship (robust regression line shown in gray). (D) The computation described in Panel C was performed across different network wiring costs, and the resulting uncorrected *p* value is plotted. At higher network thresholds, the similarity-performance relationship disappears.

Although our analyses have suggested a higher degree of fragmentation within higher order RSNs and a disproportionate amount of connector hubs in the data-driven “mover” nodes during memory encoding and retrieval, an important question remains regarding whether these changes in functional connectivity patterns account for variance in memory retrieval performance. We first computed the similarity between whole-brain networks, which assess global connectivity (Figure 6A). Here, we examined the differences between spatial and temporal *encoding* (overall group connectivity matrices shown in Figure 6B, see Supporting Information Figure SI 4 for the overall group connectivity matrices for spatial and temporal retrieval). We calculated the similarity between each individual’s unthresholded spatial and temporal networks and plotted the result versus their overall retrieval performance (Figure 6C). Interestingly, retrieval performance showed a significant negative relationship with functional connectivity network similarity patterns during encoding (*r* = −0.63, *p* = 0.005). Thus, the more dissimilar task-related connectivity was during encoding, the better an individual performed, analogous to a form of pattern separation (Yassa & Stark, 2011). This effect survived over a substantial range of network thresholds (Figure 6D, uncorrected for multiple comparisons). However, when the networks become more sparsely connected at higher threshold costs (greater than 0.8), this effect diminished, potentially indicating that some weaker and distributed connections might be providing important information involved in encoding processes. This result also held when we compared NMI between the patterns of connectivity for spatial versus temporal encoding (*r* = −0.4779, *p* = 0.0449), and thus were unlikely to be driven by differences in strengths of connections between encoding and retrieval. When investigating the other potential network contrasts, the similarity values did not correlate with participant performance when corrected for multiple comparisons (spatial retrieval vs. temporal retrieval: *r* = 0.21, *p* = 0.40; spatial encoding vs. spatial retrieval: *r* = 0.20, *p* = 0.43; temporal encoding vs. temporal retrieval: *r* = −0.23, *p* = 0.36).

**Figure 7.**
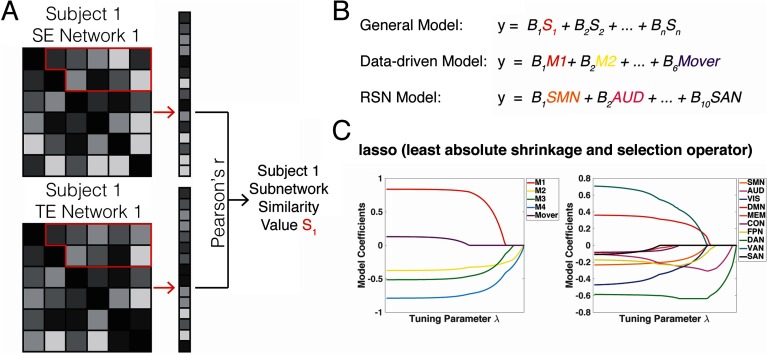
Comparison of data-driven versus resting-state models. (A) Schematic shows a theoretical Subject 1’s connectivity matrices for two conditions A and B. The unique values in the subnetwork (outlined in red) are vectorized and correlated with each other using Pearson’s *r*. This produces a single value for subnetwork similarity to assess how close the connectivity patterns are within subnetworks. (B) The multiple linear regressions are outlined where subnetwork similarity values are the independent variables and overall retrieval performance is the dependent variable (general model). For the data-driven model, the data-driven modules identified in Figure 2 constitute each variable, and the RSN networks are each variable for the RSN model. (C) By applying a variable selection technique via lasso (least absolute shrinkage and selection operator), variable coefficients in the data-driven (left panel) and RSN (right panel) regression models are reduced to zero as the tuning parameter increases, leaving a final variable that accounts for the most variance in participant performance (M4 and DAN). SE = spatial encoding; TE = temporal encoding; FPN = fronto-parietal; CON = cingulo-operculo; SAN = salience; DAN = dorsal attention; VAN = ventral attention; DMN = default mode; SMN = sensorimotor; AUD = auditory; VIS = visual; MEM = memory.

We hypothesized that there might be specific subsets of the task-related functional connectivity matrix influencing this relationship with retrieval performance. We constructed two multiple linear regressions where the similarity between two subnetworks constituted each independent variable, and subnetworks were defined either by the task-related data-driven or RSN partitions described earlier. Figure 7A provides a schematic of the subnetwork similarity calculation. Figure 7B outlines the two multiple linear regression models using the two partitions. The data-driven model was significant ($R_{adjusted}^{2}$ = 0.54, *p* = 0.01) and a better predictor of performance compared with the model based on RSN partitions ($R_{adjusted}^{2}$ = 0.23, *p* = 0.31), even when accounting for the difference in degrees of freedom between the two partitions (ΔAIC = 9.33). Again, when investigating the other potential network contrasts (spatial retrieval vs. temporal retrieval, spatial encoding vs. spatial retrieval, and temporal encoding vs. temporal retrieval), none of the multiple linear regression models were significant when corrected for multiple comparisons and regardless of partition. Notably, the data-driven correlation with performance was more parsimonious overall, likely because there were fewer independent variables compared with the full RSN model (5 compared to 10 total subnetworks).

To understand the single module that is the best predictor of performance, we next performed a variable selection technique on the two models from Figure 7B called the least absolute shrinkage and selection operator (lasso). With increasing penalty from tuning parameter, *λ*, each regression coefficient is reduced to zero until none are left in the model (Figure 7C). Here, the task-derived data-driven module “M4” (comprised of FPN, MEM, and DAN nodes) and the dorsal attention network “DAN” from the RSN model remained in terms of explaining significant amounts of variance in performance (note: FPN, MEM, and DAN all derive from partitions from the Power-based atlas). M4 and DAN served as negative predictors of performance (see Figure 7C, left and right panels, respectively), consistent with our finding of a negative correlation between network similarity and overall network connectivity patterns during encoding. Both the *reduced* task-derived data-driven and the *reduced* RSN linear models (i.e., a simple linear regression with the single lasso-selected variable as the independent variable) accounted for significant but comparable amounts of variance in performance (data-driven: $R_{adjusted}^{2}$ = 0.40, *p* = 0.003; RSN: $R_{adjusted}^{2}$ = 0.48, *p* = 0.0009; ΔAIC = 2.34) by decreases in similarity during encoding of spatial versus temporal source information. Thus, while the task-based model, as a whole, provided a better predictor of individual retrieval performance than the RSN partition (ΔAIC = 9.33), when reduced to a specific module consisting of a much smaller number of nodes, both task-driven and RSN modules explained comparable amounts of variance in retrieval performance. This, in turn, suggests that the data-driven partition, as a whole, had a slight advantage at the macroscale, but that this dissipated at a finer, node-level scale.

## DISCUSSION

In the current study, we attempted to understand the nature of task-related, large-scale functional connectivity patterns during memory encoding and retrieval. Specifically, we investigated flexible network partitions evoked during an episodic memory task for different types of context, spatial and temporal. We compared these partitions with a null baseline condition in which we expected little memory-related activity and with a widely used partition derived largely from resting-state functional connectivity patterns, the Power atlas. While RSNs are predictive of numerous individual differences and behavioral outcomes (Vaidya & Gordon, 2013), we provide evidence that task-based network architecture is also important for understanding the contributions of particular regions and their interactions specific to episodic memory processes. Specifically, we detected flexible community and individual node reorganization across multiple task states (spatial encoding, temporal encoding, spatial retrieval, temporal retrieval). We also identified distinct states during the encoding of spatial and temporal information that were associated with better performance during retrieval and leveraged community structure to explain this variance in behavior. By using a flexible, data-driven approach, we aimed to provide a more comprehensive view of the changes that can occur across the brain for many functional network interactions during the specific periods of encoding and retrieving of episodic memories.

### Contributions from Data-Driven and Resting-State Approaches

Previously, Power et al. (2011) extracted 13 RSNs (11 of which were used here for comparison) based primarily on resting-state functional connectivity and a smaller number of task-related univariate ROIs derived from a meta-analysis. In contrast, we derived our community structure based on functional connectivity patterns present during memory encoding and retrieval, which are assumed to be more closely representative of the underlying network architecture. Our community structure analyses resulted in four stable modules consisting of those nodes in the network that retained a similar connectivity pattern across conditions (spatial and temporal encoding and retrieval) and a separate group of nodes that changed communities across conditions (i.e., the “movers”). This finding persisted despite the fact that we used a resolution parameter (an input to the community detection algorithm) that typically results in a set of communities similar in number to that of RSNs (see Methods section of Cohen & D’Esposito, 2016). While the Power atlas clearly captured some of the variance related to memory encoding and retrieval in our dataset, the data-driven partition captured additional variance in community structure that was not apparent based on this atlas alone. Additionally, the mover hubs, identified based on our data-driven partitions, provide a potentially novel perspective on the flexibility of certain hubs during memory processing.

Our novel description of “mover” nodes in particular provides new insight into the flexible nature of certain nodes in memory processing. These mover nodes changed their allegiance between subnetworks consistently across encoding and retrieval of different contextual information and included both high and lower strength connections. What was the function of these mover nodes in our task? We did not find any correlation between connectivity profiles of these “moving” nodes and memory performance. Nonetheless, the tendency of these nodes to move could not be accounted for by univariate activation or by only variability among weak connections. These movers were composed of mostly connector hubs and satellite nodes, which have high participation coefficients and are important for inter-subnetwork connectivity. Because these mover nodes included areas like the hippocampus and parahippocampal gyrus that have been strongly associated with memory processing in past studies, we speculate that that flexibility in connectivity may play an integrative role for different cognitive substrates relevant in some form to memory processing. Thus, it may be that that other cognitive systems have a strong role in modulating different memory outcomes, for example during encoding versus retrieval, an idea that has been explored increasingly in the memory literature (Cabeza et al., 2018; Keerativittayayut et al., 2018; Kim et al., 2017; King et al., 2015; Schedlbauer et al., 2014). At present, the role of these mover nodes remains unclear and necessitates greater study.

### Macroscale Network Organization

Defining exact structure-function relationships in the context of memory has been difficult because many regions are active at different times and to different degrees even within or between tasks (Inman et al., 2017; Poldrack et al., 2012; Schedlbauer, & Ekstrom, 2017). Existing literature would suggest that by applying methods that capture the more flexible aspects of networks, we might better be able to understand brain-behavior relationships that correspond to particular cognitive processes (Bassett et al., 2013; Cohen & D’Esposito, 2016). In one such study, Bassett et al. (2013) tracked community structure over time during the acquisition of a new motor skill. They found a stable “core” composed of sensorimotor and visual areas that exhibited little change to their module affiliations and a flexible “periphery” composed of multimodal association areas (flexibility was defined as the frequency a brain region changed its allegiance to different communities over time). The study presented here showed similar findings, where subnetworks that were associated with primary sensory areas (SMN, AUD, VIS in Figure 3) were more stable and showed the least amount of fragmentation overall across task conditions.

In addition, Bassett and colleagues revealed that community organization indexed by the separation between the core and periphery predicted learning success. Of note, those nodes located in the periphery tended to have weaker connections. As indicated in Figure 6D, with increasing wiring cost (or the removal of weaker connections), the spatial and temporal encoding similarity-performance relationship diminished, suggesting that those weaker connections provide important information during learning. These subtle effects encourage the consideration of the efficacy of weak brain connections in support of or to the detriment of general cognitive functioning (Betzel et al., 2016; Santarnecchi et al., 2014). Furthermore, this core-periphery perspective has been similarly described in other investigations of the “rich club” phenomena, where those nodes that are highly connected tend to be mutually interconnected (van den Heuvel & Sporns, 2013). This core or rich club of nodes is thought to provide the structural and functional foundation of the brain and constitutes the main avenue of communication for the network; damage to this central structure and to the hubs belonging to it could result in widespread cognitive repercussions (e.g., Warren et al., 2014). Functional activity necessary for behavior is thought to proceed from this infrastructure where non–rich club nodes contribute to diverse behaviors.

Although resting-state analyses provide an empirically organized representation of brain connectivity, Cabeza and Moscovitch (2013) have offered an additional way to conceptually describe the transiently changing connectivity patterns in large-scale networks and the altered participation of regions during memory encoding and retrieval. Specifically, they have termed process specific alliances (PSAs) as small coalitions of nodes coming online to perform specific computations for the task at hand. PSAs are “flexible, temporary, and opportunistic.” Importantly, recruitment occurs in a task-dependent manner that may be biased but not wholly determined by larger, more stables networks, like RSNs (Krienen et al., 2014). Thus, the detection of four stable communities and an additional set of flexible “mover” nodes for different memory processes would also be in accordance with both these empirical and theoretical organizational views of the brain that emphasize the importance of flexibility to cognition. Future studies using methodologies like electroencephalography and magnetoencephalography could better probe the temporal relationships between communities or PSAs (Watrous et al., 2013).

### Functional Role of Individual Nodes in the Network

Focusing on the role of individual nodes in the network in which hubs are the locus of interest, recent work has begun to emphasize both the importance of the integrative and segregative architecture of large-scale networks and nodes that connect different subsystems throughout the brain (Bertolero et al., 2015; Cohen & D’Esposito, 2016; Cole et al., 2013; Power et al., 2013). These nodes tend to have higher participation coefficients, meaning that they connect disparate communities, and are thought to allow information flow through the network. To better understand the integration of information across multiple cognitive areas, we assigned every node an identity based on their within-module degree centrality and participation coefficient. Work from Cole et al. (2013) identified nodes in the FPN cognitive control network as having high global variability and participation coefficient, meaning that their connectivity was flexible across 64 task states and were considered hubs that integrated the network. By identifying flexible nodes within the network via their altered community structure, we were able to define a functional role for this subset of nodes during mnemonic processing, specifically the role of integration.

### Encoding of Distinct Contexts

The spatial and temporal elements of a recently experienced event are important to distinguishing between different episodes. For example, successful memory function critically depends on a person’s ability to differentiate between the different times he or she visits the same space (like going to a meeting for work in the conference room every week). When exploring the similarities and differences of global patterns of connectivity of the four networks derived from our study, we discovered a strong relationship between network similarity and participant performance. In particular, the less similar an individual’s spatial and temporal *encoding* networks were to each other, the better that participant performed during the retrieval session. Our data would support the idea, from the network perspective, that there is a behavioral advantage of separating distinct contextual information, represented here by different patterns of interaction. This idea of “pattern separation” is traditionally referred to in the context of the hippocampus, but it emphasizes orthogonalizing representations to ensure information does not overlap during encoding (Rolls & Kesner, 2006; Yassa & Stark, 2011). Our findings extend this concept to network organization, suggesting that dissimiliarity among connectivity patterns during encoding may relate to more distinct subsequent retrieval of different contextual information. Similarly, a recent study found that task-related connectivity patterns show high degrees of reconfiguration during high-encoding states (those associated with better subsequently retrieved information) compared with low-states (Keerativittayayut et al., 2018). These findings also reinforce the idea that dynamic reconfigurations of task-related functional connectivity patterns are important to successful memory retrieval.

### Technical Limitations

A number of technical analysis decisions were made regarding network and subnetwork construction, each with their own caveats and some of which limit the interpretations made. First, by using a data-driven approach, unique network partitions for each individual greatly increase the analysis space and make it more difficult to aggregate across individuals. Thus, we used the group-level, data-driven partition (as identified in Figure 2) to derive the subnetwork similarity metrics for the regression analyses. Second, while there are numerous techniques to partition networks, we applied a commonly used Louvain algorithm, which only partitions communities into nonoverlapping modules. However, there is the chance that communities could be overlapping and shared nodes or hubs, potentially altering the identity of a node within the network. Additional algorithms could and should be applied in the future to find the overlap of resulting partitions and to further explore other potential modular configurations. Third, eliminating negative connections (a consequence of the selected community detection algorithm) may disregard potentially relevant information (Parente et al., 2017), even though it is still common practice within the network community to only analyze positively weighted connections. For our analyses here, when examining the distribution of values of the weighted-connectivity matrices, it was shifted to more positive values, meaning that a larger portion of edges were positively weighted. Thus, we are confident that we have captured enough of the connectivity profile to accurately represent the networks, even though a small subset of negative connections were necessarily set to zero.

We note that task-related fMRI datasets are often more difficult to collect than large-sample resting-state studies, and thus our sample size is necessarily limited. Correlations across small sample sizes in the context of fMRI in particular should be taken with caution, and replication with a larger dataset is necessary to demonstrate the robustness of our findings (Yarkoni, 2009). In addition, recent research has shown that functional connectivity estimates can be confounded by task activations, thereby inflating correlations (Cole et al., 2019). In this study, we employed a beta series approach, which takes the correlations of fits between the behaviors of interest and the HRF (Rissman et al., 2004). By comparing these correlations between fits (beta values) in secondary *t*-tests, the method practically helps to remove variance that would otherwise inflate correlations if the HRF were not modeled (Rissman et al., 2004). In this way, the beta series technique, like the psychophysiological interactions (PPI), provides a variant of functional connectivity by accounting for common variance due to the HRF (Friston, 2011). However, this technique has not been thoroughly tested and contrasted with other measures of putative functional connectivity, and it would be helpful to know the extent to which other methods might converge or diverge with the beta series technique.

### Conclusion

Past research has placed a large emphasis on the contributions of intrinsic or RSNs to behavior, traits, and pathology. However, a focus on static network organization neglects characterization of networks representative of directed cognition, like the encoding and retrieval of episodic information in memory. The present data contribute to a growing literature emphasizing the flexible rearrangement of networks and highlight how we might conceptualize large-scale network organization during goal-directed behavior in light of RSNs.

## ACKNOWLEDGMENTS

We thank Jennifer Lieberman for data collection and Dr. Joy Geng for helpful discussions.

## SUPPORTING INFORMATION

Supporting information for this article is available at https://www.doi.org/10.1162/netn_a_00102.

## AUTHOR CONTRIBUTIONS

Amber Schedlbauer: Data curation; Formal analysis; Methodology; Validation; Visualization; Writing - Original Draft; Writing - Review & Editing. Arne Ekstrom: Conceptualization; Funding acquisition; Investigation; Methodology; Project administration; Resources; Software; Supervision; Writing - Original Draft; Writing - Review & Editing.

## FUNDING INFORMATION

Arne Ekstrom, National Institutes of Health (https://dx.doi.org/10.13039/100000002), Award ID: NS087527, NS076856.

## TECHNICAL TERMS

Encoding and retrieval: The processes of inputting information to create a memory trace that can be stored and of accessing that previously encoded and stored information, respectively.
Episodic memory: Memory for events that occur in a unique context specified by a distinct time (the “when”) and place (the “where”).
Functional connectivity: Coordinated activity between two brain regions as assessed by a statistical dependency between the time series.
Subnetworks: Smaller groups of functionally connected regions within the larger whole-brain network.
Resting-state networks (RSNs): Networks derived from brain activity measured using fMRI when participants are lying still with their eyes open and not performing a task.This contrasts with functional networks derived from participants actively engaged in a task.
Community detection: An algorithm that identifies clusters of nodes within a network that are more connected to each other than with other groups.
Source memory paradigm: An objective memory assessment where participants are required to identify a previously presented item and the context in which it appeared.
Graph theory: The study of graphs or networks and their attributes as assessed through different metrics.
Normalized mutual information (NMI): A metric that quantifies the similarity between two sets of clusters.
Least absolute shrinkage and selection operator (LASSO): A variable reduction technique for regression models that uses a regularization parameter to reduce over fitting.
