# Supplementary material for: Flexible network community organization during the encoding and retrieval of spatiotemporal episodic memories
Source: Netw Neurosci. 2019 Sep 1;3(4):1070–93. doi: 10.1162/netn_a_00102 (PMC6777981; doi:10.1162/netn_a_00102)
Supplement: Supplementary file 1 [file netn-03-1070-s001.pdf]

Schedlbauer, A., Ekstrom, A. (2019). Supporting information for "Flexible network community organization during the encoding and retrieval of spatiotemporal episodic memories." *Network Neuroscience 3*(4), 1070–1093.
https://doi.org/10.1162/netn_a_00102

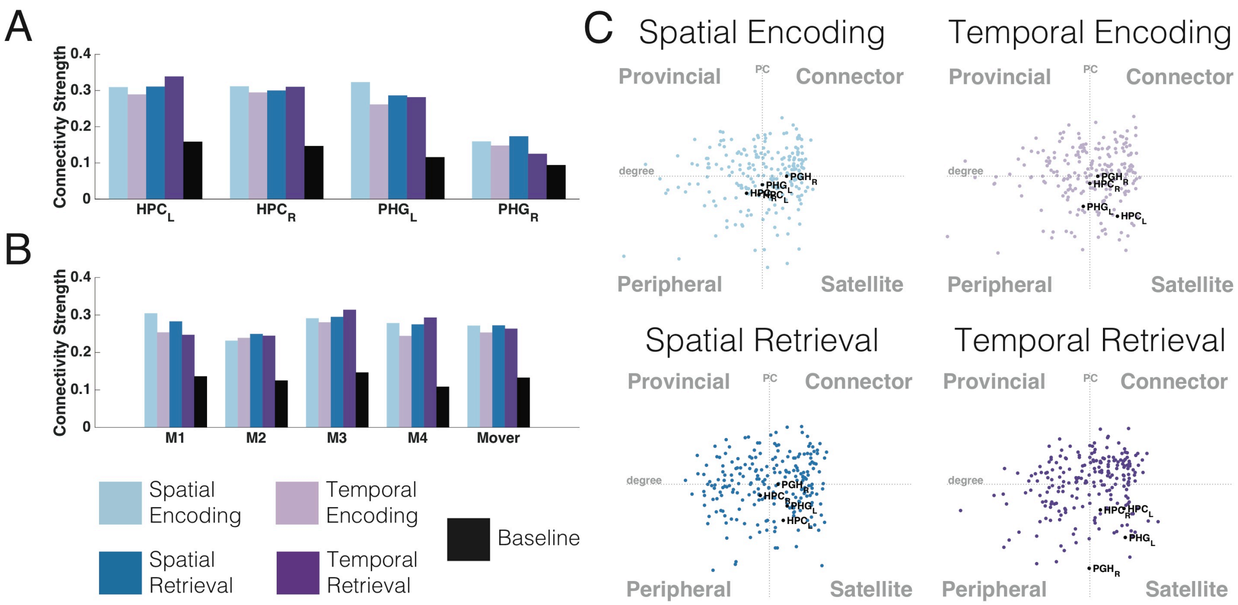

Figure SI 1: *Role of the MTL in spatiotemporal encoding and retrieval*
A) We calculated the connectivity strength of all edges connected to a single region (average edge weight) for the MTL nodes (left and right hippocampi and parahippocampal gyrii) for five conditions (spatial encoding, temporal encoding, spatial retrieval, temporal retrieval, baseline). B) We then averaged the connectivity strength for all four MTL nodes across all edges connected to the nodes belonging to the five modules identified in Figure 2 for all five conditions. C) The z-scored degree centrality and z-scored participation was calculated for all nodes in the four networks (spatial encoding – top left, temporal encoding – top right, spatial retrieval – bottom left, temporal retrieval – bottom right) and plotted, where each colored dot represents a node. We plotted the four MTL nodes in black and labeled nodes that fell into each quadrant as connector, satellite, peripheral, or provincial.

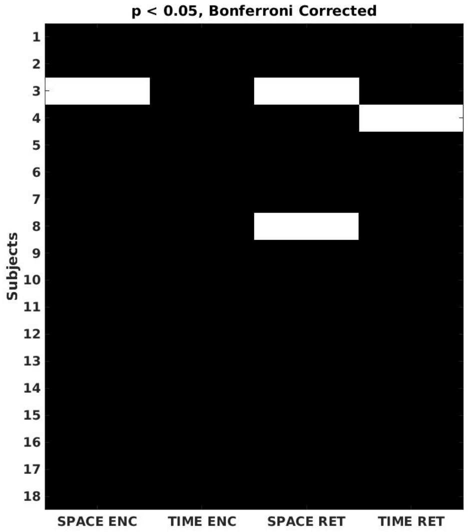

Figure SI 2: *Univariate activation compared to connectivity strength of mover ROIs*
We conducted a univariate analysis of the following contrasts for each subject: space encoding (SPACE ENC) > baseline, time encoding (TIME ENC) > baseline, space retrieval (SPACE RET) > baseline, time retrieval (TIME RET) > baseline. Then, we averaged the beta estimates across all voxels within each ROI to produce an average activity estimate for each mover region for the four conditions for each participant. We then averaged the correlation values from the individual participant adjacency matrix for each mover region, thus producing a single value for "strength of connectivity" for each mover ROI for each condition. Finally, we correlated the univariate activations for each region with the corresponding connectivity patterns for each participant and each condition. Almost none of these correlations were significant across participants (white rectangles indicate $p < 0.05$, Bonferroni corrected). The few that were significant showed no consistency by condition, possibly indicating false positives. The correlation values, when averaged across participants, were below 0.1.

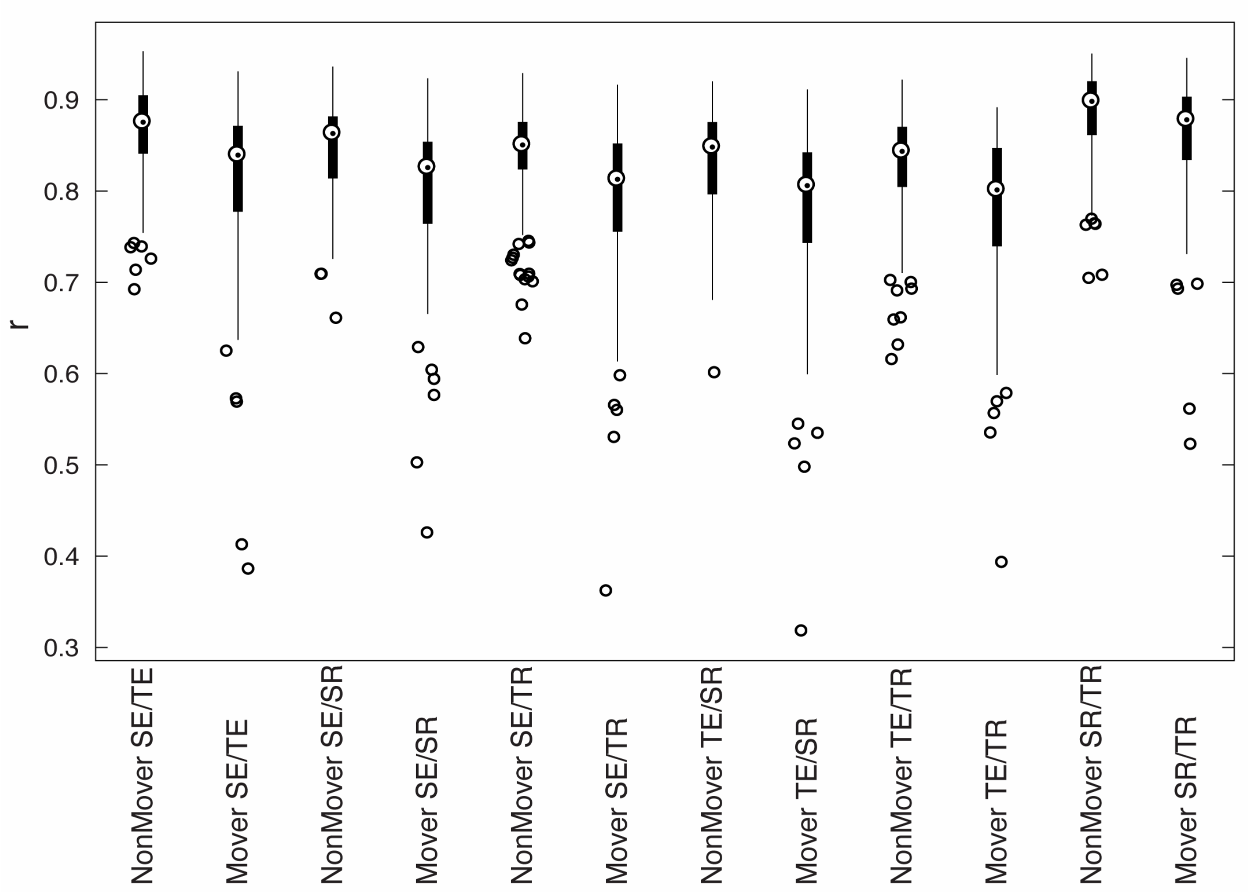

Figure SI 3: *Comparison of connectivity profiles of mover and non-mover nodes across tasks*
To see if movers significantly change the regions that they are most strongly connected to in different tasks, we correlated the connectivity vector of each node for each pairwise combination of tasks (spatial encoding (SE)/temporal encoding (TE), spatial encoding/spatial retrieval (SR), spatial encoding/temporal retrieval (TR), temporal encoding/spatial retrieval, temporal encoding/temporal retrieval, spatial retrieval/temporal retrieval) and considered the mover and non-mover nodes separately. On average, mover nodes showed a lower correlation than the non-mover nodes; this was supported by the results from a two-way ANOVA (node type X task) on the correlation (r) values derived from the connectivity vectors, which revealed a main effect of group ($F(1,1326) = 149.69$, $p < 0.001$; mover: $M = 0.80$, $SD = 0.10$, non-mover: $M = 0.85$, $SD = 0.06$). The connectivity profiles of the mover nodes were more dissimilar between tasks compared to the non-mover nodes, which suggests that those nodes are meaningfully changing their connectivity profile, rather than being an artifact of the Louvain method. In addition, there was also a main effect for task comparison ($F(5,1326) = 24.99$, $p < 0.001$), suggesting additional variability across task, but no significant interaction ($F(5,1326) = 0.89$, $p > 0.05$).

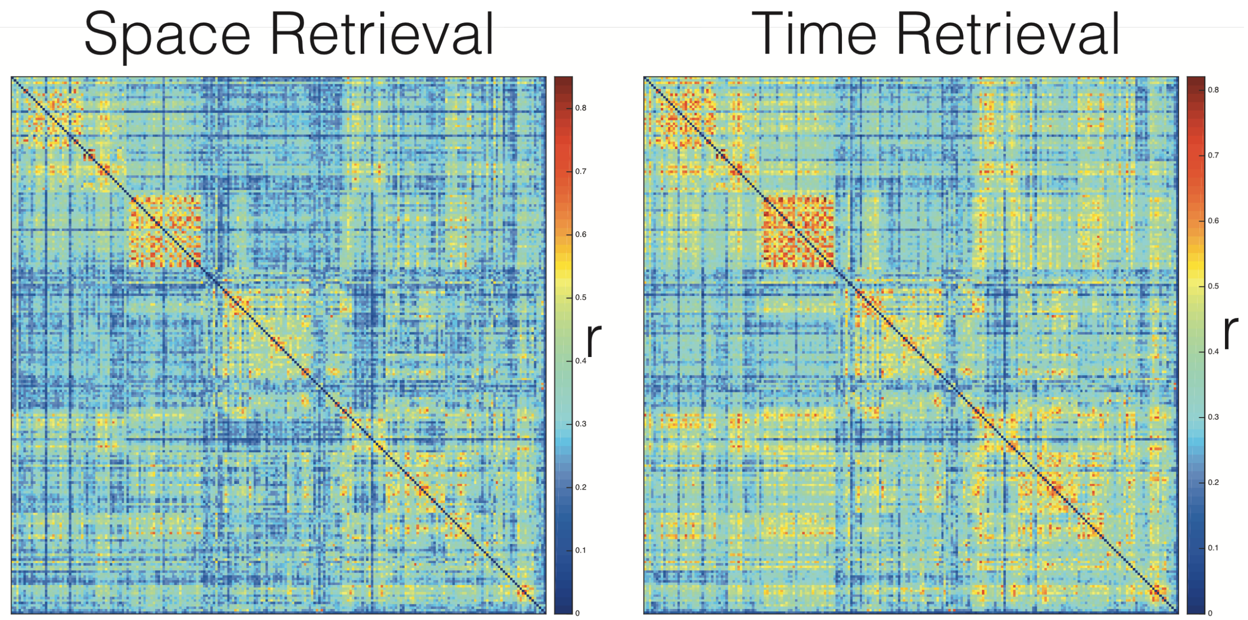

Figure SI 4: *Group-level connectivity matrices during retrieval*
The group-level connectivity matrices for the spatial retrieval (left panel) and temporal retrieval (right panel) are shown.

		Node Identity			
**RSN - AAL Location**	**Module**	Space Encoding	Time Encoding	Space Retrieval	Time Retrieval
SMN-Prec_L	M2	Satellite	Provincial	Provincial	Provincial
SMN-MCC_L	M2	Satellite	Satellite	Peripheral	Satellite
SMN-SMA_L/MCC_L/MCC_R	M2	Connector	Connector	Provincial	Provincial
SMN-SMA_R/MCC_R	M2	Peripheral	Satellite	Peripheral	Peripheral
SMN-ParaCL_L	M2	Satellite	Satellite	Peripheral	Peripheral
SMN-ParaCL_L	M2	Peripheral	Peripheral	Peripheral	Peripheral
SMN-PreCG_R/PostCG_R/ParaCL_R	M2	Provincial	Peripheral	Peripheral	Peripheral
SMN-PostCG_L/IPL_L/Smar_L	M2	Peripheral	Peripheral	Peripheral	Provincial
SMN-PreCG_R	M2	Peripheral	Peripheral	Peripheral	Peripheral
SMN-PostCG_R/Prec_R/ParaCL_R	M2	Peripheral	Provincial	Peripheral	Peripheral
SMN-PostCG_L	M2	Provincial	Provincial	Provincial	Provincial
SMN-PreCG_L/PostCG_L	M2	Peripheral	Provincial	Provincial	Provincial
SMN-PostCG_R	M2	Provincial	Provincial	Provincial	Provincial
SMN-PreCG_R/PostCG_R	M2	Provincial	Peripheral	Peripheral	Provincial
SMN-PreCG_L/PostCG_L	M2	Peripheral	Peripheral	Peripheral	Peripheral
SMN-PreCG_R	M2	Connector	Connector	Provincial	Provincial
SMN-PreCG_R/MFG_R	M2	Provincial	Provincial	Provincial	Provincial
SMN-PostCG_L/SPL_L	M2	Provincial	Provincial	Provincial	Provincial
SMN-PreCG_R/SFG_R/SMA_R	M2	Provincial	Connector	Peripheral	Peripheral
SMN-PostCG_R/SPL_R	M2	Provincial	Peripheral	Provincial	Provincial
SMN-PostCG_L/IPL_L	M2	Connector	Provincial	Provincial	Connector
SMN-PreCG_L/PostCG_L	M2	Provincial	Provincial	Provincial	Provincial
SMN-PreCG_L/ParaCL_L	M2	Provincial	Provincial	Provincial	Provincial
SMN-PreCG_R/PostCG_R	M2	Provincial	Provincial	Provincial	Peripheral
SMN-PreCG_L	M2	Peripheral	Peripheral	Peripheral	Peripheral
SMN-PostCG_L/SPL_L/Prec_L	M2	Peripheral	Peripheral	Peripheral	Peripheral
SMN-SMA_R/ParaCL_L/ParaCL_R	M2	Provincial	Provincial	Provincial	Provincial
SMN-SMA_L/SMA_R	M2	Provincial	Provincial	Provincial	Provincial
SMN-PreCG_R	M2	Provincial	Provincial	Provincial	Provincial
SMN-PostCG_R/IPL_R	M2	Connector	Peripheral	Provincial	Provincial
SMN-PostCG_L	M2	Peripheral	Peripheral	Peripheral	Peripheral
SMN-RolOper_R/Insula_R	M2	Peripheral	Satellite	Peripheral	Peripheral
SMN-PreCG_R/PostCG_R	M2	Provincial	Peripheral	Provincial	Peripheral
SMN-PostCG_L	M2	Peripheral	Peripheral	Peripheral	Peripheral
SMN-PostCG_R	M2	Peripheral	Peripheral	Peripheral	Peripheral
AUD-Insula_R/Heschl_R	M2	Connector	Connector	Provincial	Connector
AUD-STG_R	Mover	Connector	Satellite	Provincial	Provincial
AUD-RolOper_R/STG_R	M2	Connector	Connector	Provincial	Provincial

AUD-RolOper_L	M2	Connector	Connector	Provincial	Provincial
AUD-RolOper_L/Smar_L/STG_L	M2	Provincial	Provincial	Provincial	Provincial
AUD-STG_L	M2	Connector	Connector	Provincial	Connector
AUD-RolOper_R	M2	Peripheral	Peripheral	Peripheral	Peripheral
AUD-Smar_L	M2	Satellite	Satellite	Peripheral	Peripheral
AUD-PostCG_L/Smar_L	M2	Satellite	Satellite	Peripheral	Peripheral
AUD-RolOper_L/PostCG_L/Heschl_L	M2	Peripheral	Satellite	Peripheral	Peripheral
AUD-RolOper_R	M2	Satellite	Satellite	Peripheral	Peripheral
AUD-PostCG_R/Smar_R	M2	Peripheral	Provincial	Peripheral	Peripheral
AUD-Insula_L/Heschl_L	M2	Connector	Satellite	Connector	Connector
VIS-LingG_R/FuisG_R/Cerebellum	M3	Satellite	Satellite	Satellite	Satellite
VIS-MOG_R/MTG_R	M3	Connector	Connector	Satellite	Satellite
VIS-Calc_R	M3	Connector	Connector	Connector	Connector
VIS-Calc_L	M3	Connector	Connector	Connector	Connector
VIS-MOG_L	M3	Connector	Connector	Connector	Connector
VIS-Calc_R/LingG_R	M3	Satellite	Satellite	Satellite	Satellite
VIS-SOG_L/MOG_L	M3	Satellite	Connector	Connector	Connector
VIS-LingG_R/FuisG_R	M3	Satellite	Satellite	Satellite	Satellite
VIS-LingG_L	M3	Satellite	Satellite	Connector	Connector
VIS-Calc_L	M3	Connector	Connector	Connector	Connector
VIS-IOG_R	M3	Connector	Connector	Connector	Connector
VIS-IOG_L	M3	Connector	Connector	Connector	Connector
VIS-Cuneus_L/SOG_L	M3	Satellite	Connector	Connector	Connector
VIS-Cuneus_R/SOG_R	M3	Connector	Connector	Peripheral	Connector
VIS-SOG_R/MOG_R	M3	Connector	Connector	Connector	Connector
VIS-Calc_R/LingG_R	M3	Satellite	Satellite	Satellite	Satellite
VIS-Cuneus_R	M3	Connector	Connector	Peripheral	Connector
VIS-LingG_L/Prec_L	M3	Connector	Connector	Connector	Connector
VIS-IOG_R/ITG_R	M3	Connector	Connector	Satellite	Connector
VIS-SOG_R	M3	Connector	Connector	Connector	Connector
VIS-Cuneus_R/Prec_R	M3	Connector	Connector	Satellite	Connector
VIS-MOG_L	M3	Connector	Satellite	Connector	Connector
VIS-FuisG_R/Cerebellum	M3	Connector	Connector	Connector	Connector
VIS-Cuneus_L/SOG_L	M3	Connector	Connector	Peripheral	Connector
VIS-Cuneus_L	M3	Satellite	Satellite	Satellite	Connector
VIS-MOG_L/IOG_L	M3	Satellite	Satellite	Satellite	Connector
VIS-MOG_R	M3	Connector	Connector	Connector	Connector
VIS-Calc_R	M3	Connector	Satellite	Satellite	Connector
VIS-MOG_L	M3	Satellite	Satellite	Satellite	Satellite
VIS-IOG_L/FuisG_L	M3	Connector	Connector	Connector	Connector

VIS-MOG_R	M3	Connector	Connector	Satellite	Connector
DMN-MOG_L/AngG_L	Mover	Satellite	Connector	Satellite	Satellite
DMN-mIFGOrb_R	M1	Peripheral	Satellite	Peripheral	Peripheral
DMN-mIFGOrb_R/Rectus_R	M1	Peripheral	Peripheral	Peripheral	Peripheral
DMN-HPC_L/LingG_L/Prec_L	Mover	Satellite	Satellite	Satellite	Satellite
DMN-SFG_L	Mover	Peripheral	Peripheral	Satellite	Satellite
DMN-AngG_L/MTG_L	M1	Provincial	Provincial	Provincial	Provincial
DMN-MOG_R/AngG_R	Mover	Satellite	Satellite	Satellite	Connector
DMN-mTP_L/ITG_L	M1	Peripheral	Peripheral	Peripheral	Peripheral
DMN-sTP_R/mTP_R	M1	Peripheral	Peripheral	Peripheral	Satellite
DMN-MTG_L/ITG_L	M1	Provincial	Provincial	Peripheral	Peripheral
DMN-AngG_L	M1	Provincial	Provincial	Provincial	Provincial
DMN-IPL_L/AngG_L	M1	Peripheral	Peripheral	Peripheral	Peripheral
DMN-PCC_L/Prec_L	M1	Peripheral	Provincial	Provincial	Provincial
DMN-Prec_R	M1	Provincial	Provincial	Provincial	Connector
DMN-Cuneus_L/Prec_L	Mover	Peripheral	Peripheral	Provincial	Satellite
DMN-PCC_L/Prec_L	M1	Provincial	Provincial	Connector	Connector
DMN-MCC_R/PCC_R	M1	Provincial	Provincial	Provincial	Provincial
DMN-Prec_R	Mover	Connector	Satellite	Connector	Connector
DMN-MCC_L	M1	Provincial	Provincial	Connector	Connector
DMN-Prec_R	Mover	Peripheral	Peripheral	Peripheral	Satellite
DMN-IPL_R/AngG_R	M1	Provincial	Provincial	Provincial	Provincial
DMN-SFG_R	M1	Provincial	Provincial	Provincial	Provincial
DMN-SFG_L/mSFG_L	M1	Peripheral	Peripheral	Peripheral	Peripheral
DMN-SFG_L	M1	Peripheral	Peripheral	Peripheral	Peripheral
DMN-MFG_L	M1	Peripheral	Peripheral	Peripheral	Peripheral
DMN-SFG_R	M1	Peripheral	Provincial	Connector	Peripheral
DMN-SFG_R/mSFG_R	M1	Provincial	Peripheral	Connector	Peripheral
DMN-SFG_L/mSFG_L	M1	Peripheral	Peripheral	Peripheral	Peripheral
DMN-SFG_L	M1	Provincial	Provincial	Provincial	Provincial
DMN-mSFG_R/ACC_R	M1	Provincial	Provincial	Provincial	Provincial
DMN-mSFG_R	M1	Provincial	Provincial	Peripheral	Provincial
DMN-mSFG_L/mIFGOrb_L/ACC_L	M1	Peripheral	Provincial	Peripheral	Peripheral
DMN-mSFG_R	M1	Provincial	Provincial	Provincial	Provincial
DMN-mIFGOrb_L/ACC_L	M1	Peripheral	Peripheral	Provincial	Provincial
DMN-mIFGOrb_R/ACC_R	M1	Peripheral	Peripheral	Provincial	Satellite
DMN-mSFG_L/ACC_L	M1	Peripheral	Provincial	Peripheral	Peripheral
DMN-mSFG_L	M1	Provincial	Provincial	Connector	Provincial
DMN-mSFG_L/ACC_L	M1	Provincial	Provincial	Connector	Connector
DMN-SFG_L	M1	Peripheral	Peripheral	Provincial	Peripheral

DMN-mSFG_L	M1	Peripheral	Peripheral	Peripheral	Peripheral
DMN-MTG_R	M1	Peripheral	Provincial	Provincial	Peripheral
DMN-MTG_L	M1	Provincial	Provincial	Provincial	Provincial
DMN-MTG_L	M1	Provincial	Provincial	Provincial	Provincial
DMN-MTG_R	M1	Provincial	Provincial	Connector	Provincial
DMN-MTG_L	M1	Provincial	Provincial	Provincial	Provincial
DMN-SFG_R/mSFG_R	M1	Provincial	Provincial	Peripheral	Peripheral
DMN-ACC_R	Mover	Peripheral	Peripheral	Satellite	Satellite
DMN-STG_R/MTG_R	Mover	Satellite	Satellite	Peripheral	Satellite
DMN-PHG_L	Mover	Peripheral	Satellite	Satellite	Satellite
DMN-PHG_R/FuisG_R	Mover	Peripheral	Peripheral	Satellite	Satellite
DMN-FuisG_L/ITG_L	Mover	Satellite	Satellite	Satellite	Satellite
DMN-Cerebellum	Mover	Satellite	Peripheral	Satellite	Peripheral
DMN-MTG_R/mTP_R	M1	Satellite	Satellite	Satellite	Peripheral
DMN-MTG_L	M1	Peripheral	Peripheral	Peripheral	Peripheral
DMN-AngG_R	M1	Provincial	Provincial	Connector	Provincial
DMN-MTG_L	M1	Connector	Provincial	Connector	Connector
DMN-IFGOrb_L/lOFC_L	M1	Provincial	Peripheral	Provincial	Peripheral
DMN-IFGOrb_R/lOFC_R	M1	Provincial	Peripheral	Satellite	Peripheral
MEM-MCC_L/PCC_L	M1	Peripheral	Peripheral	Connector	Connector
MEM-Prec_L	Mover	Connector	Connector	Connector	Connector
MEM-Prec_R	M4	Satellite	Connector	Connector	Connector
MEM-Prec_R	Mover	Connector	Connector	Connector	Connector
MEM-MCC_L/MCC_R	Mover	Connector	Connector	Connector	Connector
CON-SMA_L	M2	Connector	Connector	Provincial	Connector
CON-Smar_R	M2	Connector	Provincial	Peripheral	Peripheral
CON-SFG_R/SMA_R	M2	Satellite	Provincial	Provincial	Provincial
CON-SFG_L	M2	Connector	Connector	Provincial	Provincial
CON-MCC_L	M2	Satellite	Satellite	Peripheral	Peripheral
CON-Putamen_R	Mover	Satellite	Satellite	Satellite	Satellite
CON-SFG_R/SMA_R	M2	Satellite	Satellite	Peripheral	Peripheral
CON-SMA_R	Mover	Connector	Connector	Connector	Connector
CON-RolOper_L/Insula_L	M2	Satellite	Satellite	Peripheral	Peripheral
CON-IFGOper_R/RolOper_R/Insula_R	Mover	Satellite	Satellite	Peripheral	Peripheral
CON-Insula_L	Mover	Satellite	Connector	Peripheral	Provincial
CON-IFGOper_L/RolOper_L/STG_L/sTP_L	Mover	Connector	Connector	Provincial	Provincial
CON-MCC_L	Mover	Connector	Connector	Connector	Connector
CON-Insula_R	Mover	Satellite	Satellite	Peripheral	Satellite
FPN-PreCG_L	Mover	Connector	Connector	Connector	Connector

FPN-IFGTri_R	Mover	Connector	Provincial	Connector	Connector
FPN-IFGOper_L/IFGTri_L	M4	Connector	Connector	Connector	Connector
FPN-IPL_L	M1	Provincial	Provincial	Connector	Provincial
FPN-SFG_L/MFG_L	Mover	Satellite	Peripheral	Satellite	Satellite
FPN-ITG_R	M4	Connector	Connector	Connector	Connector
FPN-aOFC_R	Mover	Satellite	Satellite	Satellite	Satellite
FPN-MFG_R/aOFC_R	Mover	Satellite	Peripheral	Satellite	Peripheral
FPN-PreCG_R/IFGOper_R	Mover	Connector	Connector	Connector	Connector
FPN-PreCG_L/MFG_L	Mover	Connector	Provincial	Connector	Satellite
FPN-MFG_L/IFGTri_L	Mover	Connector	Connector	Connector	Connector
FPN-MFG_R	Mover	Connector	Connector	Connector	Connector
FPN-IPL_R/Smar_R	Mover	Satellite	Satellite	Satellite	Connector
FPN-SPL_L/IPL_L	M4	Connector	Connector	Connector	Connector
FPN-IPL_R	Mover	Provincial	Satellite	Connector	Connector
FPN-SFG_R/MFG_R	Mover	Provincial	Provincial	Connector	Connector
FPN-SOG_R/MOG_R/AngG_R	Mover	Satellite	Satellite	Connector	Connector
FPN-IPL_L/AngG_L	Mover	Provincial	Provincial	Connector	Connector
FPN-MFG_R	Mover	Provincial	Peripheral	Satellite	Provincial
FPN-SFG_L/MFG_L	Mover	Provincial	Peripheral	Satellite	Provincial
FPN-MFG_L/IFGOrb_L	Mover	Peripheral	Peripheral	Satellite	Provincial
FPN-IPL_R/AngG_R	M4	Satellite	Satellite	Satellite	Connector
FPN-MFG_R/IFGOrb_R	Mover	Provincial	Peripheral	Satellite	Connector
FPN-MFG_L/IFGTri_L	Mover	Satellite	Provincial	Satellite	Connector
FPN-SMA_L/mSFG_L	Mover	Provincial	Provincial	Satellite	Connector
DAN-Prec_R	Mover	Satellite	Connector	Connector	Connector
DAN-MTG_L	Mover	Connector	Connector	Provincial	Connector
DAN-SOG_R/SPL_R/AngG_R	Mover	Satellite	Connector	Connector	Connector
DAN-MTG_R	Mover	Connector	Connector	Provincial	Satellite
DAN-SPL_R	Mover	Connector	Connector	Connector	Connector
DAN-IPL_L	M4	Satellite	Satellite	Satellite	Connector
DAN-SOG_L/MOG_L/IPL_L	M4	Connector	Connector	Connector	Connector
DAN-PreCG_L/MFG_L	M4	Satellite	Satellite	Connector	Connector
DAN-IOG_L/ITG_L	M3	Satellite	Connector	Connector	Connector
DAN-SPL_L/Prec_L	M2	Connector	Connector	Provincial	Connector
DAN-PreCG_R/SFG_R/MFG_R	M2	Connector	Connector	Provincial	Provincial
VAN-SMA_L	M1	Connector	Connector	Satellite	Connector
VAN-Smar_R/STG_R	Mover	Connector	Connector	Connector	Connector
VAN-STG_L/MTG_L	Mover	Connector	Connector	Connector	Connector
VAN-STG_L	Mover	Satellite	Satellite	Satellite	Satellite
VAN-STG_R	Mover	Satellite	Satellite	Satellite	Satellite

VAN-STG_R/MTG_R	Mover	Connector	Satellite	Satellite	Connector
VAN-STG_R/MTG_R	Mover	Connector	Connector	Connector	Connector
VAN-IFGTri_R	Mover	Connector	Connector	Satellite	Satellite
VAN-IFGTri_L	M1	Provincial	Provincial	Peripheral	Provincial
SAN-MCC_R/Prec_R/ParaCL_R	Mover	Connector	Connector	Provincial	Provincial
SAN-IPL_R/Smar_R	Mover	Satellite	Connector	Satellite	Connector
SAN-PreCG_R	Mover	Connector	Connector	Connector	Connector
SAN-MFG_R	Mover	Connector	Connector	Connector	Connector
SAN-IFGOper_R/IFGTri_R	Mover	Satellite	Satellite	Satellite	Satellite
SAN-IFGTri_L/Insula_L	Mover	Satellite	Satellite	Satellite	Satellite
SAN-Insula_R	Mover	Satellite	Satellite	Satellite	Satellite
SAN-IFGTri_R/IFGOrb_R/Insula_R	Mover	Satellite	Satellite	Satellite	Satellite
SAN-Insula_R	Mover	Peripheral	Peripheral	Satellite	Satellite
SAN-ACC_L	Mover	Satellite	Satellite	Satellite	Satellite
SAN-SMA_L/mSFG_L/MCC_L	Mover	Connector	Connector	Connector	Connector
SAN-SFG_L/MFG_L	Mover	Connector	Provincial	Connector	Connector
SAN-ACC_L/ACC_R/MCC_R	Mover	Connector	Connector	Connector	Connector
SAN-mSFG_L/MCC_L/MCC_R	Mover	Connector	Connector	Connector	Connector
SAN-ACC_R/MCC_R	Mover	Satellite	Satellite	Satellite	Satellite
SAN-SFG_R	Mover	Connector	Connector	Satellite	Satellite
SAN-SFG_R/MFG_R	Mover	Connector	Connector	Connector	Connector
SAN-MFG_L	Mover	Satellite	Satellite	Satellite	Satellite
MEM-HPC_L	Mover	Peripheral	Satellite	Satellite	Satellite
MEM-HPC_R	Mover	Peripheral	Satellite	Peripheral	Satellite
MEM-PHG_L	Mover	Peripheral	Satellite	Satellite	Satellite
MEM-PHG_R	Mover	Connector	Connector	Connector	Peripheral

SI Table 1: *Node locations, modular assignment, and identity*
Each row contains the assigned resting-state network from the Power Atlas and anatomical location from the AAL atlas (column 1), the data-driven module assignment (column 2), and the node identity for spatial encoding (column 3), temporal encoding (column 4), spatial retrieval (column 5), and temporal retrieval (column 6) for all 223 nodes in the networks.
